# Genome-Wide Identification of the *LdARF* Gene Family in *Lilium davidii* var. *unicolor* and Transient Functional Analysis of *LdARF17* in Bulblet Regeneration

**DOI:** 10.3390/plants15172581

**Published:** 2026-08-24

**Authors:** Ying Tang, Yiqing Wang, Shuxin Gong, Wenjie Guo, Shuting Zhang, Shaozhong Fang, Xiaoping Xu, Chenglong Yang, Zhongxiong Lai

**Affiliations:** 1 Institute of Horticultural Biotechnology, Fujian Agriculture and Forestry University, Fuzhou 350002, China; yingtang0408@163.com (Y.T.); g2035878113@163.com (S.G.); shutingvictory@yeah.net (S.Z.); 2 Fujian Provincial Key Laboratory of Agricultural Genetic Engineering, Biotechnology Research Institute, Fujian Academy of Agricultural Science, Fuzhou 350003, China; 18305921379@163.com (Y.W.); gwj73@126.com (W.G.); fangshaozhong1969@163.com (S.F.); byxxp310107@163.com (X.X.); 3 Beijing Key Laboratory of Development and Quality Control of Ornamental Crops, Department of Ornamental Horticulture, China Agricultural University, Beijing 100193, China

**Keywords:** Auxin response factor, *Lilium davidii* var. *unicolor*, bulblet regeneration, *LdARF17*

## Abstract

Auxin response factors (ARFs) are key transcriptional regulators of the auxin signaling pathway and play important roles in plant organogenesis and regeneration. However, the functions of ARF family genes in lily scale-derived bulblet regeneration remain largely unclear. In this study, 24 *LdARF* genes were identified from the genome of *Lilium davidii* var. *unicolor*. Phylogenetic analysis revealed that LdARF proteins showed evolutionary conservation with ARF homologs from other monocot species. Genome-wide identification, phylogenetic analysis, and expression profiling revealed functional divergence among *LdARF* genes during scale-derived bulblet regeneration. Among them, *LdARF17* exhibited a distinct regeneration-associated expression pattern, characterized by rapid induction after scale excision and sustained high expression during subsequent bulblet initiation and formation. Subcellular localization analysis demonstrated that LdARF17 is localized in the nucleus. Transient overexpression of *LdARF17* significantly promoted bulblet regeneration and was associated with increased expression of auxin-responsive and regeneration-related genes, including *IAA14*, *LBD16*, and *LBD29*. These findings suggest that *LdARF17* acts as a positive regulator of lily scale regeneration and may influence auxin-responsive transcriptional processes associated with early cell proliferation, providing new insights into the molecular mechanisms underlying vegetative regeneration in lilies.

## 1. Introduction

Plant regeneration is a highly coordinated developmental process controlled by complex hormonal and transcriptional regulatory networks. Among phytohormones, auxin plays a central role in regulating cell dedifferentiation, proliferation, and organ formation during regeneration [1,2,3]. During wound-induced regeneration, local auxin accumulation activates cell division, induces regeneration-related gene expression, and promotes the establishment of new meristematic tissues, ultimately leading to organogenesis [3,4,5].

Auxin response factors (ARFs) are key transcriptional regulators that mediate auxin signaling by controlling downstream gene expression. In *Arabidopsis*, *ARF7* and *ARF19* regulate lateral root formation and callus induction by activating LATERAL ORGAN BOUNDARIES DOMAIN (*LBD*) genes such as *LBD16* and *LBD29*, functioning as central regulators of organ regeneration [6,7]. In addition, ARFs interact with WUSCHEL-related homeobox (WOX) transcription factors to regulate cell fate reprogramming and promote the formation of adventitious organs [8,9]. Together with PIN-mediated auxin transport, ARF-dependent transcriptional regulation contributes to the establishment of spatial auxin responses and tissue patterning during regeneration [10,11,12]. Similar ARF-mediated regulatory modules have been identified in rice, maize, and other plant species, indicating the conserved roles of ARFs in regeneration processes [13,14,15,16,17,18]. Moreover, comparative analyses of ARF gene families across horticultural plants have demonstrated that ARF proteins are evolutionarily conserved while exhibiting species-specific diversification patterns [19].

Lanzhou lily (*Lilium davidii* var. *unicolor*) is a perennial bulbous plant with both medicinal and edible value and is widely cultivated in China due to its large bulbs, high nutritional quality, and considerable economic importance [20,21,22]. As an important edible lily germplasm resource, it plays a key role in regional horticultural production and the bulb industry. However, large-scale propagation of Lanzhou lily relies predominantly on vegetative reproduction through bulb division and scale-derived bulblet regeneration [23]. In practice, this regeneration process is often inefficient and highly variable, which severely limits commercial production and breeding efficiency. This limitation has become a major bottleneck in the industrial utilization of Lanzhou lily. During scale propagation, regeneration efficiency is influenced by multiple factors, including the physiological status of donor scales, pathogen infection, and environmental conditions [24,25]. Notably, scale explants exhibit strong regenerative potential at their basal regions, where adventitious bulblets can be induced, making them an ideal experimental system for studying plant regeneration mechanisms [25,26,27].

Previous transcriptomic and physiological studies have demonstrated that hormone signaling pathways, especially auxin-related processes, are closely associated with lily bulblet initiation and development. For example, exogenous auxin treatment was shown to promote bulblet formation by modulating auxin-responsive genes and hormone-related pathways in lily [28]. In addition, transcriptome analyses and functional studies have identified several transcription factors, such as *LOB* domain-containing proteins, that participate in the regulation of lily bulblet formation [29]. Despite these advances, the upstream transcriptional regulators that coordinate auxin responses during lily regeneration remain largely unknown, and the specific roles of ARF family members in lily vegetative regeneration have not been systematically characterized. Furthermore, the large and complex genome of lily, together with extensive expansion of gene families, has hindered the functional characterization of key regulatory genes [30,31,32].

Therefore, identifying ARF members involved in lily regeneration and elucidating their potential regulatory roles will provide new insights into the molecular mechanisms underlying bulblet regeneration. In this study, we performed a genome-wide identification and systematic characterization of the *ARF* gene family in *Lilium davidii* var. *unicolor*. The expression profiles of *LdARF* genes were analyzed throughout different stages of scale-derived bulblet regeneration, and *LdARF17* was identified as a regeneration-associated candidate gene. Subcellular localization and transient transformation assays were performed to investigate the potential role of *LdARF17* in bulblet regeneration. Furthermore, the expression patterns of auxin-responsive and regeneration-related genes, including members of the *IAA*, *LBD*, *WOX*, and *PIN* gene families, were analyzed to explore the possible regulatory pathways associated with *LdARF17* during lily bulblet regeneration.

## 2. Results

### 2.1. Identification, Chromosomal Distribution, and Physicochemical Properties of the LdARF Gene Family

A total of 24 *LdARF* genes were identified in the *L. davidii* var. *unicolor* genome (Table 1). These genes were unevenly distributed across 12 chromosomes, with the highest number observed on chromosome LG05 (five members). Based on chromosomal localization, the genes were designated as *LdARF1–LdARF24* (Figure S1). The predicted protein lengths of LdARF members ranged from 370 to 1144 amino acids (aa). The predicted instability index values of LdARF proteins ranged from 44.80 to 73.40. The theoretical isoelectric points (pI) ranged from 5.16 to 9.81, with 18 acidic proteins (pI < 7) and six basic proteins (pI > 7), indicating that the majority of LdARF proteins exhibit acidic characteristics. The aliphatic index ranged from 65.77 to 87.17, and the GRAVY values were negative (−0.750 to −0.165), suggesting that LdARF proteins are hydrophilic. Subcellular localization prediction showed that 16 LdARF proteins were predicted to be localized in the nucleus (nucl), while the remaining members were predicted to be localized in the chloroplast (chlo), cytoplasm (cyto), endoplasmic reticulum (ER), peroxisome (pero), vacuole (vacu), and Golgi apparatus (golg). These results suggest potential differences in the subcellular localization of LdARF proteins, which may contribute to their functional diversification.

### 2.2. Conserved Motifs, Domain Architecture, and Gene Structure of LdARF Proteins

**Figure 1:**
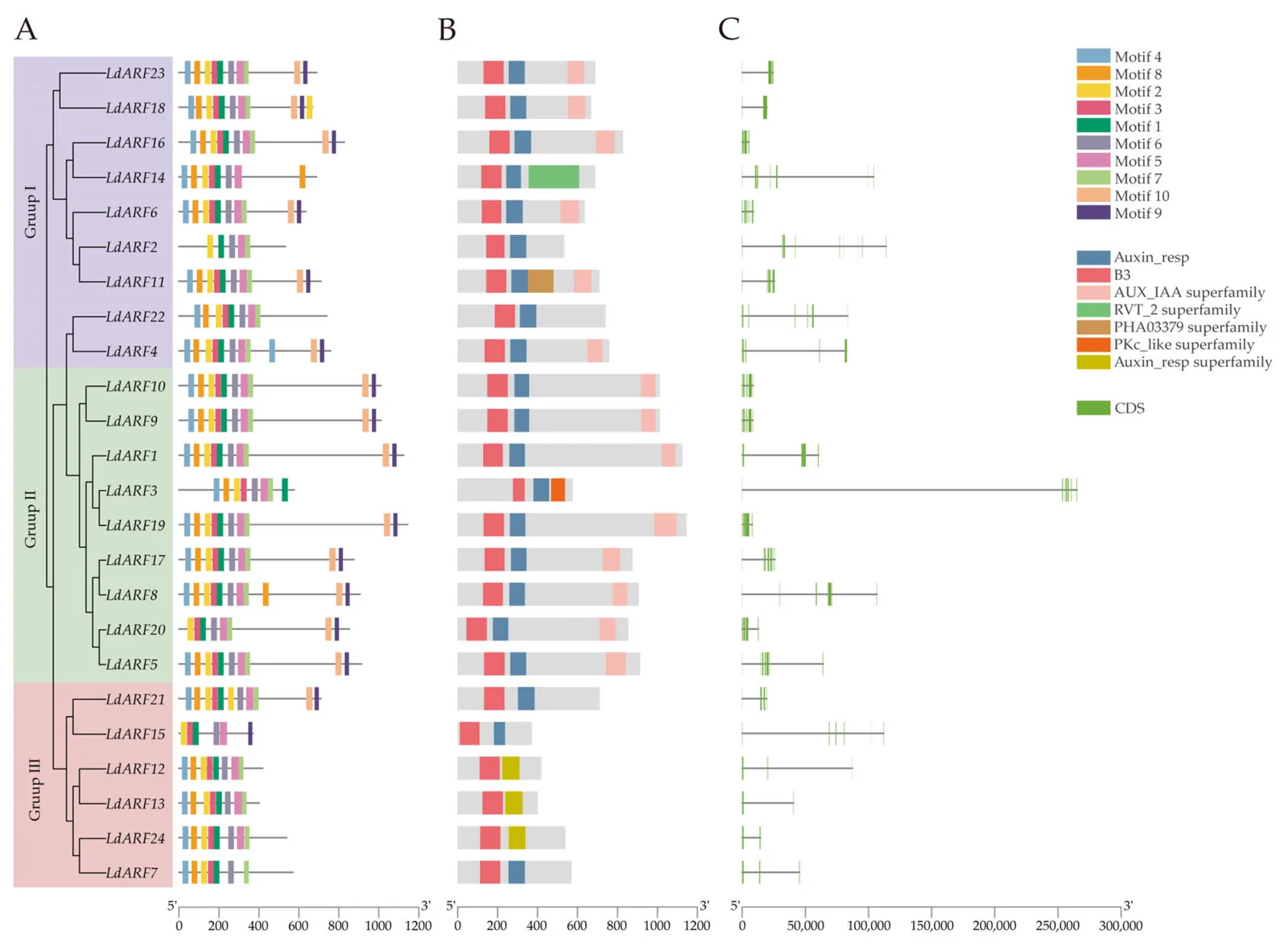
Conserved motif composition, domain architecture, and gene structure analysis of *LdARF* family members. (**A**) Motif composition of LdARF proteins. (**B**) Domain architecture of LdARF proteins. (**C**) Gene structure of *LdARF* genes.

Phylogenetic relationships, conserved motifs, protein domains, and gene structures of LdARF proteins were analyzed (Figure 1). Motif analysis showed that Motif 1, Motif 2, and Motif 6 were present in all LdARF members. Members within the same phylogenetic clade exhibited similar motif composition and arrangement patterns, while differences were observed between clades. Some members, including *LdARF2*, *LdARF3*, and *LdARF15*, showed variation in motif composition, with some motifs being absent or additionally detected. Domain analysis indicated that all LdARF proteins contained a conserved B3 domain. Twenty-one LdARF proteins contained an Auxin_resp domain, whereas three members (*LdARF12*, *LdARF13*, and *LdARF24*) were annotated only with an Auxin_resp superfamily domain. The AUX/IAA domain was predominantly detected in LdARF proteins belonging to Group I and Group III. Furthermore, exon–intron structure analysis showed substantial differences in gene length and intron distribution among the 24 *LdARF* members. Members clustered within the same phylogenetic group displayed relatively conserved exon–intron organization, whereas genes from different groups showed distinct structural patterns. Among them, *LdARF3* exhibited the largest gene length, mainly characterized by an extremely long intronic region, resulting in a distinct genomic structure compared with other *LdARF* genes.

### 2.3. Phylogenetic Analysis and Synteny of the LdARF Gene Family

**Figure 2:**
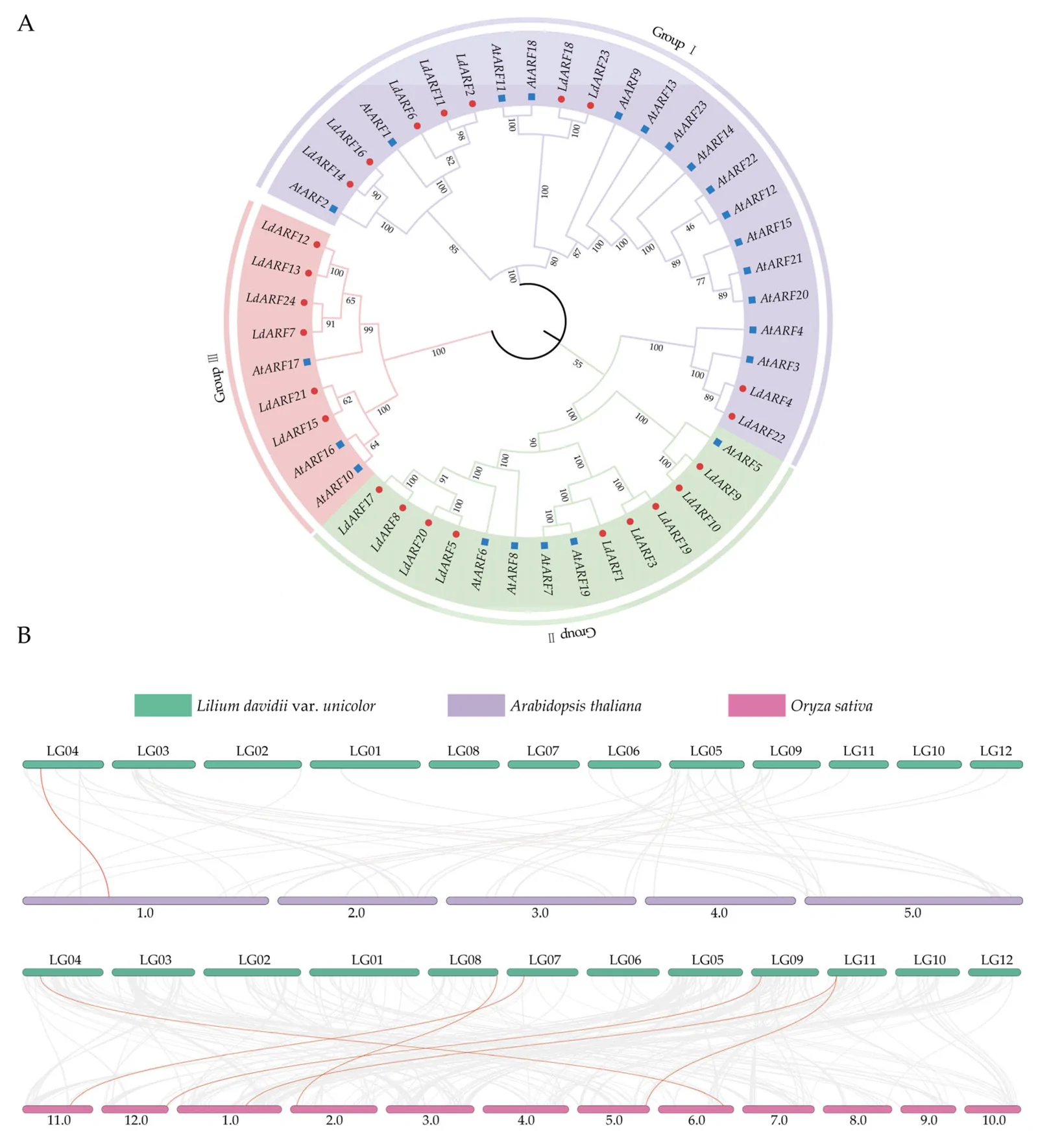
Phylogenetic and synteny analysis of the *LdARF* gene family. (**A**) Phylogenetic tree of LdARF proteins and AtARF proteins. Red dots represent LdARF proteins, and blue squares represent AtARF proteins. (**B**) Synteny analysis of ARF genes among *A. thaliana*, *O. sativa*, and *L. davidii* var. *unicolor*. Different species are indicated by different colors, and orange lines represent orthologous gene pairs.

A phylogenetic tree was constructed using the neighbor-joining (NJ) method based on aligned ARF protein sequences from *A. thaliana* and *L. davidii* var. *unicolor* (Figure 2A). According to the classification of the model species *A. thaliana*, the ARF proteins were divided into three subfamilies, designated Group I, Group II, and Group III. The 24 LdARF proteins were unevenly distributed among these subfamilies, with Group I and Group II containing nine members each, and Group III containing six members.

To investigate the evolutionary relationships and potential expansion mechanisms of the *LdARF* gene family, synteny analysis was performed between *Lilium davidii* var. *unicolor*, *Arabidopsis thaliana*, and *Oryza sativa* (Figure 2B). Syntenic blocks were detected between lily and both reference species, indicating the presence of conserved syntenic relationships among ARF genes across species. In the *L. davidii* var. *unicolor* genome, *LdARF* genes were distributed across 12 linkage groups (LG01–LG12), showing a broad chromosomal distribution pattern. In addition, six orthologous gene pairs were identified between lily and rice, suggesting conserved evolutionary relationships between ARF genes in these two species.

Furthermore, intraspecific synteny analysis was performed to investigate duplication events within the *L. davidii* var. *unicolor* genome (Figure S2). One segmental duplication pair was identified among the *LdARF* genes, corresponding to *LdARF7* and *LdARF12*, whereas no tandem duplication events were detected. To evaluate the evolutionary pressure acting on this duplicated gene pair, Ka/Ks analysis was conducted. The Ka/Ks ratio of the *LdARF7*–*LdARF12* duplicated gene pair was calculated as 0.276, which was lower than 1, indicating that this duplicated gene pair has evolved under purifying selection.

### 2.4. Cis-Acting Regulatory Elements in the Promoters of LdARF Genes

**Figure 3:**
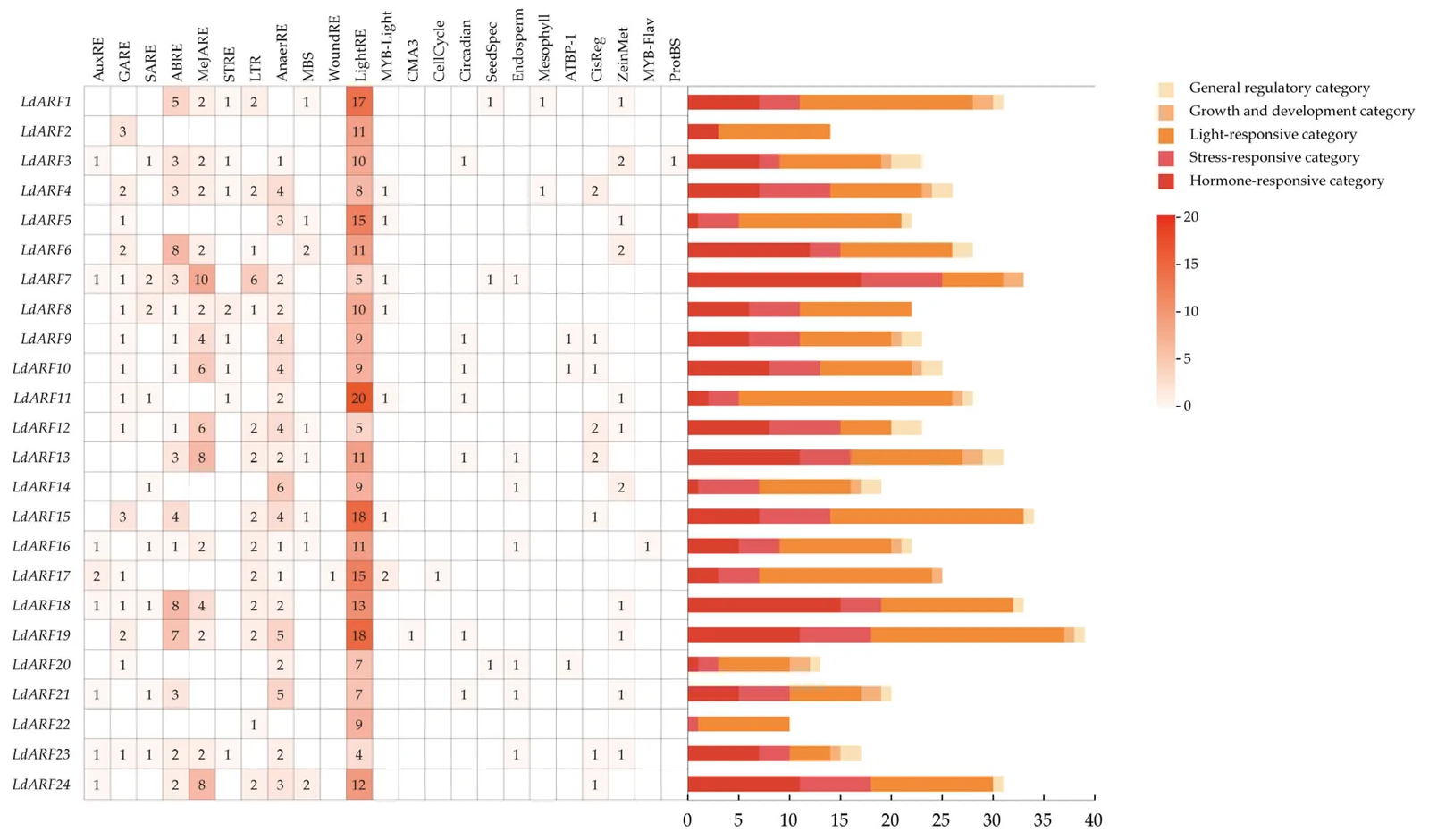
Cis-acting regulatory elements in the promoters of *LdARF* genes. Red labels and numbers indicate the abundance of each cis-acting regulatory element in *LdARF* promoters.

The 2000 bp upstream promoter regions of *LdARF* genes were extracted and analyzed for cis-acting regulatory elements (Figure S3). To visualize the types and distribution of predicted cis-elements, all identified elements were classified and quantitatively summarized (Figure 3). The results showed that multiple types of regulatory elements were present in the promoters of *LdARF* genes, which were categorized into five major groups, including hormone-responsive, light-responsive, stress-responsive, growth and development-related, and general regulatory elements. Light-responsive elements were highly abundant in the promoters of all *LdARF* genes. In addition, hormone-responsive elements such as AuxRE, GARE, and ABRE were widely detected in the promoter regions, indicating the presence of multiple hormone-associated regulatory components. Stress-related elements, including W-box, LTR, STRE, and MBS, were also identified in most *LdARF* promoters. Quantitative analysis based on stacked bar plots showed that the total number of cis-elements varied among *LdARF* genes, ranging from approximately 10 to over 30. Different *LdARF* members displayed distinct compositions and proportions of cis-element categories. Some genes exhibited dominance of a single category, whereas others contained multiple categories of regulatory elements. Overall, variation in the composition and abundance of cis-acting elements was observed among *LdARF* family members, indicating diversity in promoter architecture.

### 2.5. Expression Patterns of the LdARF Gene Family

**Figure 4:**
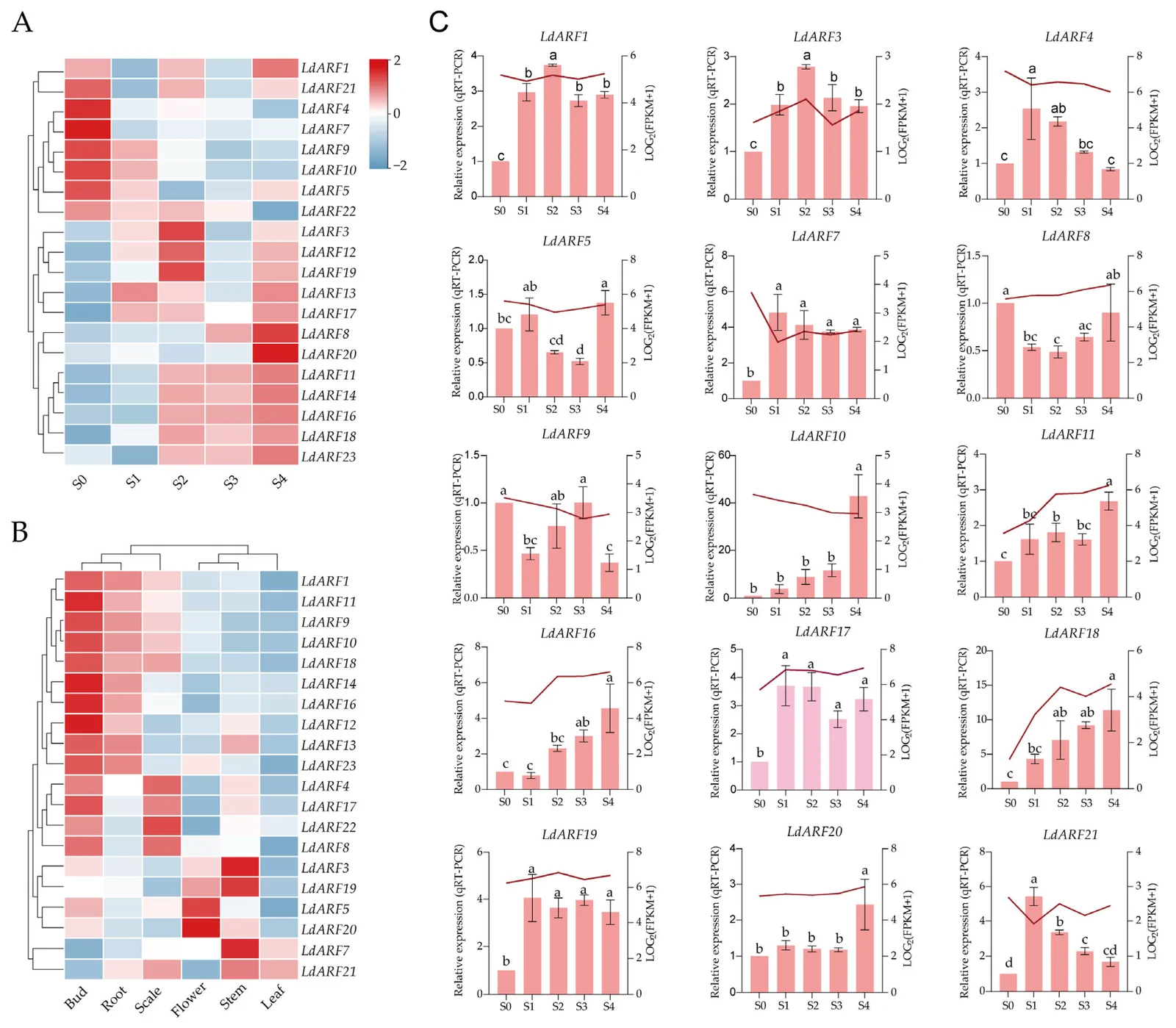
Expression patterns of *LdARF* genes during scale-derived bulblet regneration and in different tissues. (**A**) Expression profiles of *LdARF* homologs across bulblet regeneration stages. Red color indicates higher expression levels. (**B**) Tissue-specific expression profiles of *LdARF* homologs in root, stem, leaf, flower, bud, and scale. (**C**) qRT-PCR analysis of selected *LdARF* genes in *Lilium davidii* var. *unicolor* during bulblet regeneration. Different lowercase letters (e.g., a, b, c) above the bars indicate significant differences according to Tukey’s multiple comparison test (*p* < 0.05); letters are compared within each individual gene across S0–S4 time points. RNA-seq data in panels A and B were obtained from the ‘Siberia’ transcriptome dataset, whereas qRT-PCR analysis in panel C was performed in *L. davidii* var. *unicolor*. S0 indicates the time of scale excision; S1, 3 days after planting; S2, 9 days after planting with slight swelling at the basal region; S3, visible meristem-like structures; S4, fully developed bulblets or adventitious roots.

Transcriptome data from five developmental stages (S0–S4) of scale-derived bulblet regeneration in the cultivar ‘Siberia’, together with multiple tissues (root, stem, leaf, flower, bud, and scale), were used to analyze ARF homolog expression profiles corresponding to the *LdARF* gene family. Because a reference genome of ‘Siberia’ is currently unavailable, these transcriptome datasets were annotated using the published *L. davidii* var. *unicolor* genome as the reference (Tables S2 and S3). Although derived from different lily genetic backgrounds, the ‘Siberia’ transcriptome dataset was used as a reference for identifying regeneration-associated ARF expression patterns, and candidate genes were further validated in *L. davidii* var. *unicolor* by qRT-PCR (Figure 4C and Figure S4, Table S4). Tissue-specific expression analysis revealed that ARF homologs of the *LdARF* genes were broadly expressed across different organs. Buds exhibited the highest number of highly expressed genes, whereas scales and stems showed moderate expression levels, and roots and leaves contained relatively fewer highly expressed members (Figure 4B). In scale tissues, homologs corresponding to *LdARF4*, *LdARF8*, *LdARF17*, and *LdARF22* showed relatively higher expression levels. During scale-derived bulblet regeneration, ARF homologs displayed distinct stage-dependent expression patterns (Figure 4A,C). A subset of genes, including *LdARF1*, *LdARF3*, *LdARF7*, *LdARF17*, and *LdARF19*, showed increased expression during early stages (S1–S2). Among them, the *LdARF17* homolog exhibited a marked increase in expression during early regeneration and reached its highest expression level at S2–S3 stages. By contrast, the expression levels of *LdARF10*, *LdARF11*, *LdARF16*, and *LdARF18* exhibited a sustained increase throughout the process. Expression of *LdARF4* and *LdARF21* gradually decreased during S1–S4, while *LdARF5*, *LdARF8*, *LdARF9*, and *LdARF20* showed fluctuating expression patterns.

Among the identified *LdARF* genes, *LdARF17* displayed a distinct regeneration-associated expression pattern characterized by rapid induction after scale excision and sustained expression during early bulblet initiation and formation. This temporal expression pattern was observed in the ‘Siberia’ transcriptome dataset and was further supported by qRT-PCR analysis in *L. davidii* var. *unicolor*. Phylogenetic analysis revealed that *LdARF17* clustered within the *AtARF6/8* subgroup, which contains ARF members involved in auxin-mediated developmental regulation. In addition, promoter analysis revealed that the upstream regulatory region of *LdARF17* contains multiple predicted auxin-responsive elements (AuxREs) and wound-responsive cis-elements, suggesting its potential involvement in responses to auxin and wound-related signaling during regeneration. Based on the combined evidence from its distinct regeneration-associated expression dynamics, evolutionary relationship, and predicted regulatory characteristics, *LdARF17* was selected as a candidate gene for further functional characterization.

### 2.6. LdARF17 Silencing Negatively Affects Bulblet Regeneration in Lily Scales

**Figure 5:**
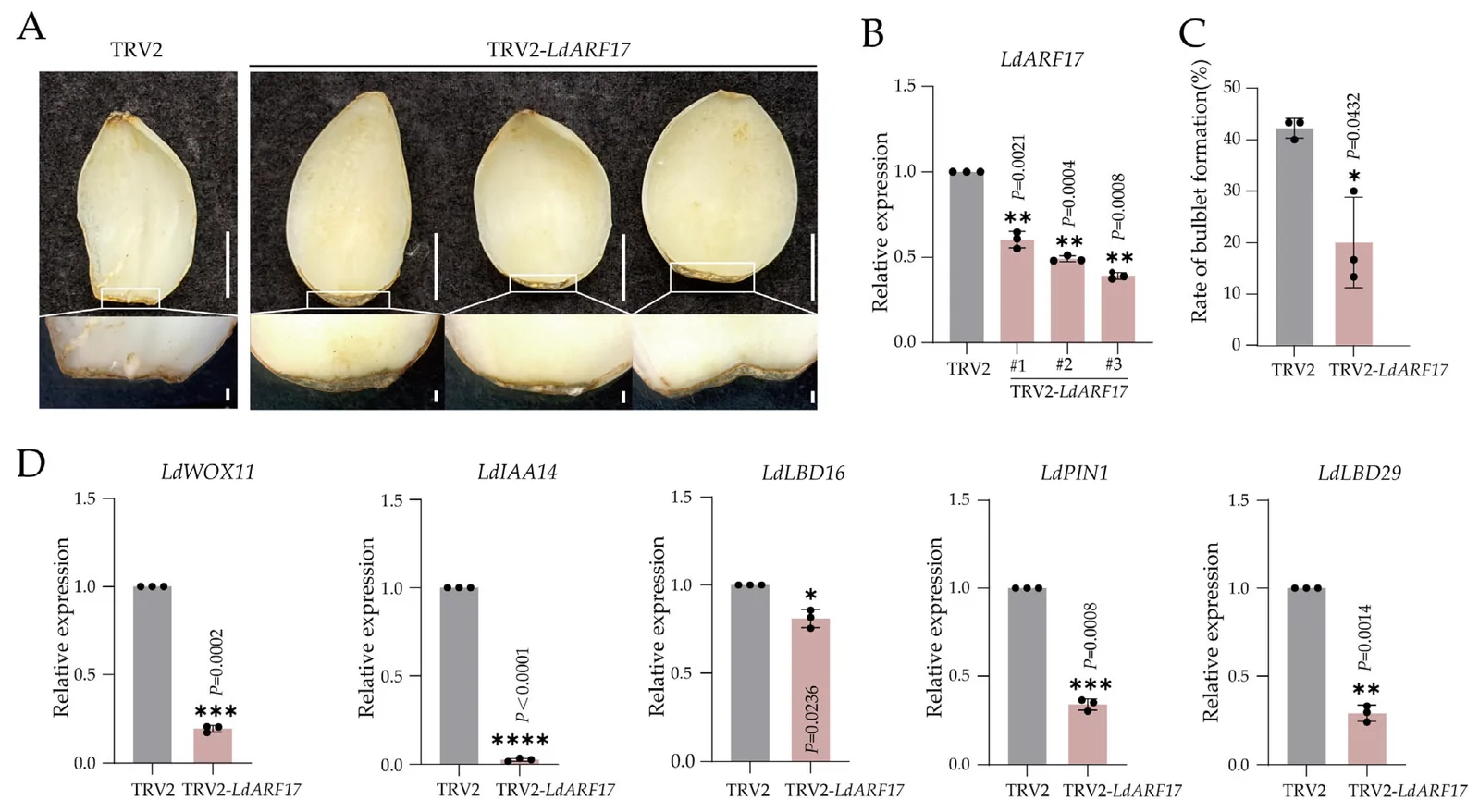
Virus-induced gene silencing (VIGS) of *LdARF17* suppresses bulblet formation in lily scales. Both the TRV2 empty vector control group and TRV2-*LdARF17* silencing group were set up with three biological replicates (*n* = 3). (**A**) Phenotypic comparison of scale explants infiltrated with TRV2 (control) and TRV2-*LdARF17*. Scale bars = 1 cm and 1 mm. (**B**) Relative expression levels of *LdARF17* in control and silenced samples. (**C**) Bulblet formation rate under TRV2 and TRV2-*LdARF17* treatments. Formation rate was calculated as the number of regenerated scales divided by the total number of cultured scales. (**D**) Expression levels of bulblet- and auxin-related genes, including *WOX11*, *IAA14*, *LBD16*, *PIN1*, and *LBD29*. Data are presented as mean ± SD. Statistical significance was determined using one-way ANOVA followed by Dunnett’s multiple comparisons test for panel B, and two-tailed Student’s *t*-test for panels (**C**,**D**). * *p* < 0.05, ** *p* < 0.01, *** *p* < 0.001, **** *p* < 0.0001.

To further explore the potential role of *LdARF17* in bulblet regeneration, a TRV2-*LdARF17* construct was generated and introduced into scale explants via *Agrobacterium*-mediated infiltration. PCR detection of the CP (coat protein) gene produced a specific band of the expected size in treated samples, indicating that the viral vector had successfully entered the plant tissues (Figure S4). To evaluate potential off-target effects, the expression levels of three randomly selected ARF family members (*LdARF3*, *LdARF9*, and *LdARF21*) were examined by qRT-PCR. These genes did not show a consistent reduction pattern across independent TRV2-*LdARF17* silencing lines (Figure S4). Compared with the control group (TRV2), *LdARF17*-silenced explants showed a reduction in bulblet formation at the basal region of scales, although the extent of the phenotype varied among independent biological replicates (Figure 5A–C). qRT-PCR analysis showed reduced expression levels of *LdARF17* in silenced tissues and revealed altered expression patterns of several auxin- and regeneration-related genes (Figure 5D). Expression levels of *IAA14*, *LBD16*, *LBD29*, *WOX11*, and *PIN1* were all reduced in the silencing group. Among these, *IAA14* showed the strongest reduction, whereas *LBD16* displayed a moderate decrease.

### 2.7. Transient Overexpression of LdARF17 Promotes Bulblet Regeneration in Lily Scales

**Figure 6:**
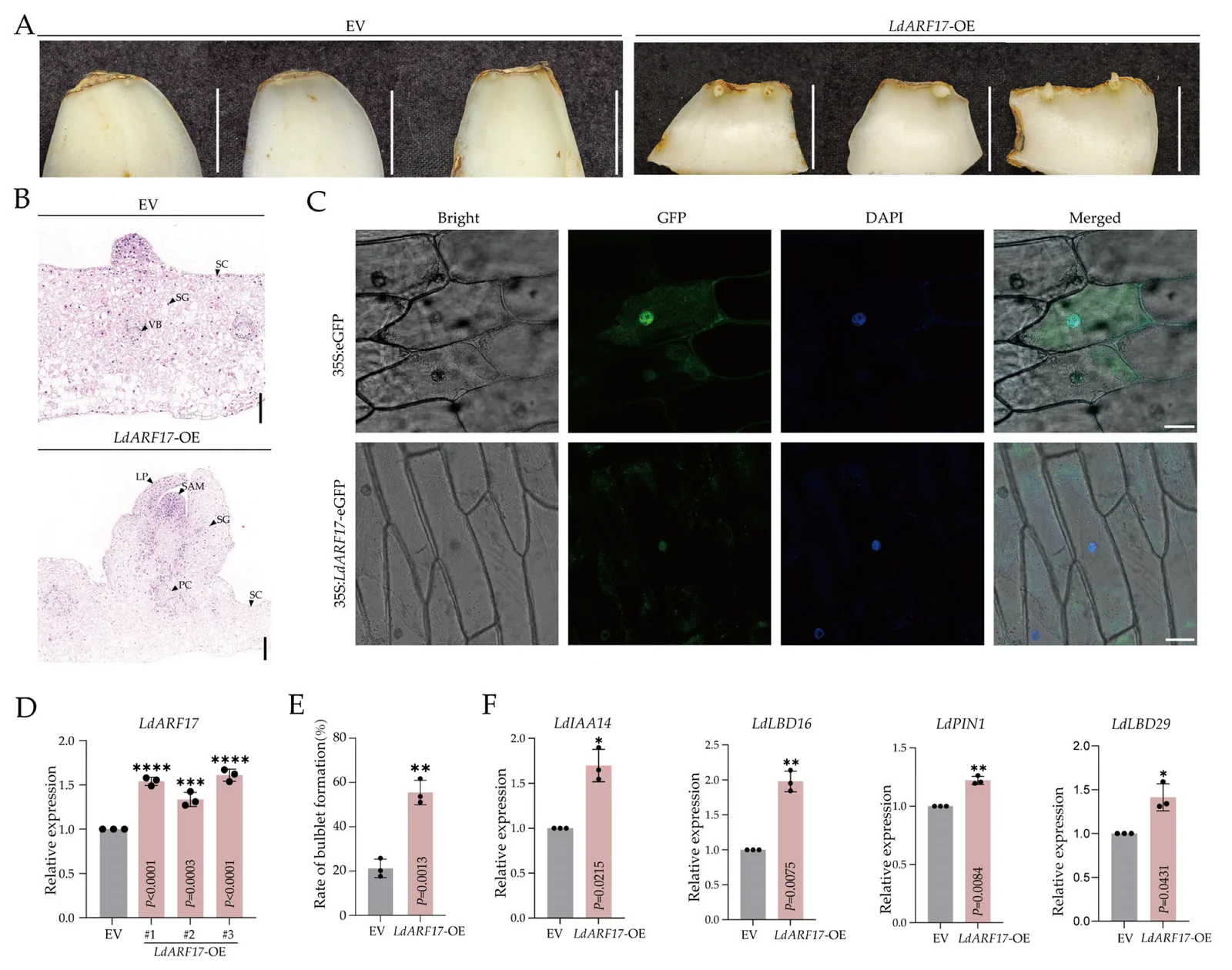
Overexpression and subcellular localization of *LdARF17* in lily scales. The empty vector control (EV) group and *LdARF17* overexpression (OE) group were established with three biological replicates (*n* = 3). (**A**) Phenotypic comparison of lily scale explants infiltrated with empty vector (EV) and *LdARF17* overexpression (*LdARF17*-OE). Scale bar = 1 cm. (**B**) Histological analysis of bulblet formation using paraffin sections stained with hematoxylin-eosin (HE). Black arrows indicate different tissue structures. SC, scale epidermal cell; SG, starch granule; VB, vascular bundle; PC, parenchymal cell; SAM, shoot apical meristem; LP, leaf primordium. Scale bars = 200 μm and 250 μm. (**C**) Subcellular localization of LdARF17 in onion epidermal cells. Green fluorescence indicates the eGFP-LdARF17 fusion protein, and nuclei were stained with DAPI. Bright-field images are shown for reference. Scale bar = 75 μm. (**D**) Relative expression level of *LdARF17* in EV and overexpression samples. (**E**) Bulblet formation rate under EV and *LdARF17*-OE treatments. Formation rate was calculated as the number of regenerated scales divided by the total number of cultured scales. (**F**) Relative expression levels of four target genes (*IAA14*, *LBD16*, *LBD29*, and *PIN1*). Data are presented as mean ± SD. Statistical significance was determined using one-way ANOVA followed by Dunnett’s multiple comparisons test for panel (**D**), and two-tailed Student’s *t*-test for panels E and F. * *p* < 0.05, ** *p* < 0.01, *** *p* < 0.001, **** *p* < 0.0001.

A p35S::*LdARF17*::GUS overexpression construct was generated and introduced into lily scale explants via vacuum infiltration, with empty vector (EV, p35S::GUS) serving as the control. Regeneration performance was evaluated 15 days after infiltration, and bulblet formation was statistically analyzed. PCR amplification confirmed the presence of GUS and Hyg fragments in transformed tissues, indicating successful introduction of the recombinant construct (Figure S6). qRT-PCR analysis further showed significantly elevated expression of *LdARF17* in three independent OE lines compared with the EV control (Figure 6D). Phenotypic observation revealed that overexpression of *LdARF17* promoted bulblet formation at the basal region of scale explants compared with the control (Figure 6A). Histological analysis revealed more pronounced bulblet primordia formation at the basal region of OE scales, accompanied by increased starch granule accumulation compared with the EV control (Figure 6B). Quantitative analysis further demonstrated a significant increase in bulblet formation frequency in the OE group (Figure 6E). Expression analysis of auxin- and regeneration-related genes showed upregulation of *IAA14*, *LBD16*, *PIN1* and *LBD29* in *LdARF17*-overexpressing tissues (Figure 6D,F).

### 2.8. Subcellular Localization Analysis of LdARF17

Subcellular localization analysis in onion epidermal cells showed that LdARF17 was predominantly localized in the nucleus (Figure 6C). In cells expressing the pCAMBIA2300-eGFP-LdARF17 fusion protein, green fluorescence signals were observed mainly in the nucleus and overlapped with DAPI-stained nuclear signals. In contrast, free eGFP signals in the control were distributed throughout both the cytoplasm and nucleus, showing no specific localization pattern.

## 3. Discussion

### 3.1. Evolutionary Conservation and Functional Divergence of the LdARF Gene Family in Lily

Auxin response factors (ARFs) are key transcriptional regulators that mediate auxin-responsive gene expression and participate in diverse aspects of plant growth and development. The typical ARF protein structure, consisting of an N-terminal B3 DNA-binding domain, a middle regulatory region, and a C-terminal Auxin_resp domain, provides the structural basis for auxin-dependent transcriptional regulation [2,33,34]. Although this conserved architecture is generally maintained among plant species, sequence divergence and structural variation among individual ARF members may contribute to functional specialization during plant evolution.

In this study, 24 *LdARF* genes were identified in *Lilium davidii* var. *unicolor* (Table 1), with a family size comparable to those reported for *A. thaliana* and *O. sativa* [35,36,37]. Phylogenetic analysis classified LdARF proteins into three major clades, consistent with the classical classification of ARF members in *A. thaliana* (Figure 2A). The clustering of lily ARF members with their corresponding *Arabidopsis* ARF homologs within the same phylogenetic branches supports the hypothesis that the major evolutionary lineages of ARFs were established early in plant evolution and have been conserved throughout the diversification of monocots and dicots.

Despite the conserved phylogenetic framework, structural diversification was observed among LdARF proteins. Several LdARFs, including *LdARF12*, *LdARF13*, and *LdARF24*, exhibited variations in their Auxin_resp domain annotations, suggesting potential differences in protein regulatory properties (Figure 1B). Similar atypical ARF members have been identified in *A. thaliana* and other plant species [38,39,40]. For example, *Arabidopsis* ARF3/ETTIN lacks the canonical C-terminal PB1 domain that mediates interaction with Aux/IAA proteins, but retains its function in regulating floral organ development, indicating that divergent domain architectures do not necessarily impair ARF activity and may contribute to alternative regulatory mechanisms [41]. Therefore, the atypical domain organizations observed in several LdARF proteins may reflect evolutionary diversification within the ARF family and contribute to functional diversity among ARF members. However, whether these divergent LdARF proteins retain canonical ARF activities or possess specialized regulatory functions remains to be experimentally determined.

Motif composition and gene structure analyses further supported functional diversification of *LdARF* genes (Figure 1A,C). Members within the same phylogenetic clade generally showed similar motif arrangements and exon–intron organization, whereas considerable structural variation was observed between different clades. Similar patterns have been reported in rice, maize, and other plant species, suggesting that gene duplication followed by sequence divergence has contributed to the functional differentiation of ARF family members [36,42,43]. Notably, *LdARF3* contained an exceptionally long intron, which may be associated with the accumulation of repetitive elements and genome structural expansion characteristic of the giant lily genome [31,32]. In addition, the limited number of duplication events, conserved syntenic relationships, and low Ka/Ks ratio of the duplicated gene pair suggest that *LdARF* family expansion was not mainly driven by recent duplication events and that duplicated ARF genes have been maintained under strong evolutionary constraints.

Promoter analysis revealed that *LdARF* genes contain diverse cis-regulatory elements associated with hormone responses, light signaling, and stress responses, consistent with previous reports in soybean and other plant species (Figure 3) [44,45]. The presence of these regulatory elements suggests that LdARFs may integrate developmental signals and environmental responses. This feature may be particularly relevant in lily scale-derived regeneration, a process involving wound responses, cellular reprogramming, and organ initiation.

Collectively, the *LdARF* family exhibits both evolutionary conservation and structural diversification. The conserved ARF domain organization provides the structural basis for the fundamental roles of LdARF proteins in auxin-responsive regulation, whereas sequence and structural variations among family members may contribute to specialized developmental functions. As a bulbous monocot with strong regenerative capacity, lily provides a valuable system for exploring the functional diversification of ARF-mediated auxin regulation during plant regeneration.

### 3.2. Dynamic Expression Patterns of LdARF Genes Reveal Their Potential Roles in Lily Bulblet Regeneration

Although LdARF members share conserved evolutionary characteristics, their distinct expression profiles indicate functional divergence among family members (Figure 4B). Transcriptome analysis revealed that *LdARF* genes displayed tissue-preferential expression patterns across buds, roots, scales, flowers, stems, and leaves, suggesting that different ARF members may participate in specific developmental processes. Similar expression divergence has been observed in monocot crops, where *OsARF* and *ZmARF* genes exhibit distinct organ-specific expression patterns associated with diverse developmental functions [36,42].

In addition to regulating normal plant development, ARFs have been widely associated with regeneration-related processes, including wound responses, cellular dedifferentiation, and organ initiation. In *A. thaliana*, *ARF6* and *ARF8* participate in adventitious root formation, whereas *ARF7* and *ARF19* regulate auxin-mediated organogenesis through downstream developmental pathways [6,46,47]. In terms of underground storage organ formation, studies in potato, carrot, and sugar beet have demonstrated that auxin signaling networks are involved in tuber initiation and expansion processes, while ARFs, as core auxin-responsive transcription factors, may participate in this regulatory mechanism [48]. These findings highlight the conserved involvement of ARF-mediated transcriptional regulation in developmental transitions.

Lily bulblet regeneration is a complex developmental process involving sequential events, including wound responses, acquisition of regenerative competence, meristem initiation, primordium formation, and bulblet enlargement [26]. Integrated transcriptome analysis and qRT-PCR validation revealed distinct temporal expression patterns among *LdARF* genes during regeneration, suggesting that different ARFs may function at specific developmental stages (Figure 4A). Early-induced LdARFs may contribute to the activation of regeneration-related responses following scale excision, whereas members with sustained or late-stage expression may participate in subsequent organ formation and bulblet development. Other dynamically expressed members may contribute to maintaining auxin signaling balance during developmental transitions.

A limitation of this study is that the transcriptome dataset used for stage-specific expression analysis was generated from the cultivar ‘Siberia’, whereas genome-based gene identification and functional analyses were performed in *Lilium davidii* var. *unicolor*. Because a reference genome for ‘Siberia’ is currently unavailable, the transcriptome reads were aligned to the published *L. davidii* var. *unicolor* genome. Although this strategy enabled the identification of homologous genes and characterization of temporal expression patterns, cultivar-specific genetic variation may influence transcript abundance. For example, *LdARF7* exhibited a significant negative correlation between the RNA-seq and qRT-PCR datasets (*r* = −0.96, *p* = 0.01), indicating an opposite expression trend between the two datasets (Table S4). Therefore, the transcriptome data were interpreted primarily as evidence of expression dynamics rather than direct quantitative comparisons between these two genetic backgrounds.

### 3.3. Transient Functional Analysis of LdARF17 Suggests Its Positive Role in Lily Bulblet Regeneration

Among the identified *LdARF* members, *LdARF17* showed a characteristic regeneration-associated expression pattern and was selected for preliminary functional characterization. Although transient expression in onion epidermal cells confirmed the nuclear localization pattern of LdARF17, this heterologous system may not fully represent its localization behavior in lily cells. Future studies using a homologous lily transformation system or co-localization with nuclear markers will provide further confirmation of LdARF17 subcellular localization. Transient manipulation of *LdARF17* expression demonstrated that silencing of *LdARF17* reduced bulblet formation efficiency (Figure 5), whereas overexpression was associated with enhanced regeneration (Figure 6). Although transient overexpression of *LdARF17* resulted in a significant increase in bulblet formation efficiency, the observed increase in *LdARF17* transcript abundance was relatively moderate. This apparent discrepancy between transcriptional activation and phenotypic output may be attributed to the characteristics of the transient expression system and the temporal dynamics of transgene expression. Because samples for qRT-PCR analysis were collected 15 days after infiltration, when bulblet regeneration phenotypes had already been established, the transcript abundance detected at this stage may not fully represent the initial expression level or potential peak accumulation of *LdARF17* during the early regeneration process [49]. In addition, although the CaMV 35S promoter has been widely used for constitutive expression in both dicotyledonous and monocotyledonous plants, its activity can vary depending on plant species, tissue type, and experimental conditions [50]. Therefore, the moderate increase in *LdARF17* transcript abundance observed in lily tissues may partly reflect the expression characteristics of the CaMV 35S promoter in this specific transient transformation system. Nevertheless, the enhanced bulblet regeneration phenotype consistently observed in transient *LdARF17*-overexpression materials supports a positive regulatory role of LdARF17 in lily bulblet regeneration.

To further explore the potential role of LdARF17 in regeneration, several regeneration-associated genes were examined. In *LdARF17*-silenced and transient *LdARF17*-overexpressing materials, the expression patterns of *PIN1*, *WOX11*, *LBD16*, *LBD29*, and *IAA14* were altered and were associated with changes in *LdARF17* expression. *WOX11* plays an important role in cellular fate transition and the establishment of regenerative competence by promoting the acquisition of meristematic characteristics [51,52]. *LBD16* and *LBD29* are auxin-responsive transcription factors involved in lateral organ development and auxin-regulated developmental processes [7,53,54]. *PIN1*-mediated polar auxin transport plays an essential role in establishing local auxin maxima required for organ initiation and meristem development [11,55,56,57]. Aux/IAA proteins are important regulators of auxin signaling, as they interact with ARF transcription factors and modulate their transcriptional activity [58,59,60,61].

The altered expression of these regeneration- and auxin-related genes suggests that LdARF17 may be associated with auxin-related regulatory processes during bulblet regeneration. However, whether these genes are directly regulated by LdARF17 or act as downstream components requires further experimental validation. Such coordinated expression changes may contribute to maintaining auxin signaling stability during the transition from cellular reprogramming to organ formation.

Overall, genome-wide identification and expression profiling revealed extensive conservation and diversification within the *LdARF* gene family. Among these members, *LdARF17* displayed a regeneration-associated expression pattern and positively influenced bulblet formation, suggesting that specific ARF members may acquire specialized functions during lily regeneration. These findings expand the understanding of ARF family evolution and provide candidate genes for further investigating auxin-mediated regulatory networks underlying lily scale-derived regeneration.

## 4. Materials and Methods

### 4.1. Plant Materials and Regeneration System

Scale-derived bulblet regeneration was induced using disease-free *L. davidii* var. *unicolor* plants. The outer 2–3 scales were removed, and the middle scales were cultured in a substrate (peat:vermiculite = 3:1, *v*/*v*) in a growth chamber at 24 °C under dark conditions. The substrate was sterilized with carbendazim prior to use.

Regeneration stages were defined as follows: S0, scale excision; S1, 3 days after planting; S2, slight swelling at the basal region (9 days); S3, visible meristem-like structures; and S4, fully developed bulblets or adventitious roots, according to the classification criteria described by Kang et al. [26]. Approximately 0.5 cm of basal tissue from each stage was collected, immediately frozen in liquid nitrogen, and stored for RNA extraction and gene expression analysis.

### 4.2. Genome-Wide Identification and Characterization of the LdARF Gene Family

ARF genes were identified from the *L. davidii* var. *unicolor* genome using a combination of BLASTP and hidden Markov model (HMM)-based searches. The protein sequences of *A. thaliana* ARF family members were used as queries for BLASTP searches against the lily protein database using the BLASTP function implemented in TBtools v2.472, with an E-value cutoff of 1 × 10^−5^. In parallel, the HMM profile of the ARF domain (PF06507) was obtained from the Pfam database and used to search the lily protein database using the Simple HMM Search function implemented in TBtools v2.472 with default parameters. Candidate ARF proteins identified by both BLASTP and HMM-based searches were retained for further analysis. The conserved domains of candidate proteins were subsequently verified using the NCBI Conserved Domain Database (CDD) and InterPro database. Proteins lacking complete ARF domains or containing incomplete ARF domains were excluded. Finally, a total of 24 *LdARF* genes were identified and used for subsequent analyses. Physicochemical properties, including amino acid length, molecular weight, and isoelectric point, were predicted using TBtools v2.472. Chromosomal locations were determined based on GFF3 annotation files, and genes were renamed according to chromosomal order [62]. Subcellular localization was predicted using WoLF PSORT.

### 4.3. Motif, Gene Structure, and Promoter Analysis

Conserved motifs were identified using MEME Suite v5.5.9 with the Classic mode. The maximum number of motifs was set to 10, with motif widths ranging from 6 to 50 amino acids. The motif occurrence model was set to zero or one occurrence per sequence (ZOOPS), and the significance of identified motifs was evaluated based on the E-values calculated by MEME. Conserved domains were analyzed using the NCBI Batch CD-Search tool. Gene structures were visualized using TBtools v2.472 based on genome annotation files. Phylogenetic relationships and structural features (motifs, domains, and gene structures) were integrated using TBtools [62]. For promoter analysis, 2000 bp upstream sequences were extracted using TBtools. Cis-acting regulatory elements were predicted using PlantCARE. Element abundance was summarized in Microsoft Excel 2021, and heatmaps were generated using TBtools [62].

### 4.4. Phylogenetic and Synteny Analyses

A neighbor-joining (NJ) phylogenetic tree was constructed using MEGA 12.0 (Pennsylvania State University, State College, PA, USA) with 1000 bootstrap replicates. Genomic synteny analysis was conducted using the *Oryza sativa* genome as a reference. The one-step MCScanX pipeline in TBtools was employed to perform genome-wide collinearity analysis between *Lilium davidii* var. *unicolor* and the two reference species, *Arabidopsis thaliana* and *Oryza sativa*, and the resulting syntenic relationships were visualized [62].

### 4.5. Expression Profiling and qRT-PCR Validation

Based on sequence homology, transcriptome data from different stages (S0–S4) of scale-derived bulblet regeneration in the cultivar ‘Siberia’, previously generated by our research group, were analyzed to investigate the temporal expression patterns of ARF homologs corresponding to *L. davidii* var. *unicolor* ARF genes. Since a reference genome of ‘Siberia’ is currently unavailable, clean reads were aligned to the published *L. davidii* var. *unicolor* reference genome using HISAT2 for splice-aware alignment. Gene expression levels were quantified based on the annotated gene models of *L. davidii* var. *unicolor*. The mapping statistics are provided in Supplementary Tables S2 and S3. Heatmaps were generated using TBtools v2.472 [62]. A total of 15 *LdARF* genes were selected for qRT-PCR analysis. Total RNA was extracted from the basal tissues of scales at five regeneration stages using the HiPure Plant RNA Mini Kit (Magen, Shanghai, China; R4151-02). First-strand cDNA was synthesized using the HiScript III All-in-one RT SuperMix kit (Vazyme, Nanjing, China; R333-01) and used as the template for qRT-PCR. qRT-PCR was performed using Taq Pro Universal SYBR qPCR Master Mix (Vazyme, Nanjing, China; Q712-02) on a QuantStudio™ 1 Plus Real-Time PCR system (Thermo Fisher Scientific, Waltham, MA, USA). Each experiment included three biological replicates and three technical replicates. Primers for *LdARF* genes, regeneration-related genes (*WOX11*, *LBD16*, and *LBD29*), and auxin-related genes (*IAA14* and *PIN1*) were designed using the GenScript online tool. *LdACTIN* was used as the internal reference gene [63]. Relative expression levels were calculated using the 2^−ΔΔCt^ method.

### 4.6. Virus-Induced Gene Silencing of LdARF17

TRV vectors were provided by the Key Laboratory of Ornamental Plants, China Agricultural University. A specific non-conserved fragment of *LdARF17* was cloned into TRV2 using BamHI and XhoI restriction sites. Recombinant constructs TRV1, TRV2, and TRV2-*LdARF17* were transformed into *Agrobacterium tumefaciens* GV3101. Two groups were established: control (TRV2) and silencing (TRV2-*LdARF17*), each with three biological replicates (30 scales per replicate). *Agrobacterium* cultures were grown in LB medium with kanamycin and rifampicin. Cells were resuspended in infiltration buffer (10 mM MgCl_2_, 200 μM acetosyringone, 10 mM MES, pH 5.6) and adjusted to OD600 = 0.8. Equal volumes of TRV1 and TRV2 constructs were mixed. Vacuum infiltration was performed at 0.8 MPa for 15 min. After infiltration, scales were incubated on moist cotton pads. Bulblet regeneration was evaluated after 15 days, and infection efficiency was confirmed by RT-PCR using TRV coat protein primers.

### 4.7. Transient Overexpression of LdARF17

The full-length ORF of *LdARF17* was cloned into the pCAMBIA1301-GUS vector under the CaMV 35S promoter (p35S::*LdARF17*::GUS). The construct was introduced into *Agrobacterium tumefaciens* GV3101. *Agrobacterium* infiltration was performed as described in Section 4.6. Two groups were established: empty vector control group (EV, p35S::GUS) and *LdARF17* overexpression group (p35S::*LdARF17*::GUS), each with three biological replicates (30 scales per replicate). Bulblet regeneration was evaluated 15 days after infiltration, and samples for gene expression analysis were collected at the same time point. Transformation was confirmed by RT-PCR detection of GUS and Hyg genes.

### 4.8. Subcellular Localization of LdARF17

The coding sequence of *LdARF17* was cloned into the pCAMBIA2300-eGFP vector using BamHI and SalI sites. The recombinant construct was introduced into *Agrobacterium tumefaciens* strain GV3101. Transient expression was performed in onion epidermal cells via *Agrobacterium*-mediated infiltration. Empty vector (eGFP) served as a control. Samples were incubated at 28 °C in darkness for 24 h, and fluorescence signals were observed 2–3 days post-infiltration using a confocal laser scanning microscope (Leica Microsystems, Wetzlar, Germany).

### 4.9. Statistical Analysis

Data were analyzed using one-way analysis of variance (ANOVA). For multi-group comparisons with a single control group, Dunnett’s multiple comparisons test was performed. For comparisons between two independent groups, two-tailed Student’s *t*-tests were used. All analyses were conducted using GraphPad Prism 10.5.0.

## 5. Conclusions

In this study, 24 *LdARF* gene family members were identified from the whole-genome data of *Lilium davidii* var. *unicolor*. Systematic analyses of chromosomal distribution, protein structure, phylogeny, cis-acting elements, and expression profiles revealed the structural diversity and potential functional differentiation of the *LdARF* family. *LdARF* genes exhibited stage-specific expression patterns during scale regeneration and were classified into four categories: early continuously upregulated, late continuously upregulated, late continuously downregulated, and fluctuating types. *LdARF17* showed dynamic expression during key regeneration stages and LdARF17 protein was localized in the nucleus. Functional analyses demonstrated that silencing of *LdARF17* inhibited scale regeneration and bulblet formation, whereas transient overexpression promoted these processes, accompanied by altered expression of regeneration-related genes. These results support the conclusion that LdARF17 acts as a positive regulator of lily scale regeneration, and that changes in *LdARF17* expression are associated with altered expression patterns of regeneration-related genes, suggesting its potential role in promoting organ regeneration. The study refines the functional framework of the *LdARF* gene family in lily and provides new insights into the regulatory mechanisms underlying bulblet regeneration, offering candidate genetic resources for future functional studies and molecular breeding in lily.

## Figures and Tables

**Table 1:** Basic characteristics of ARF gene family members in *L. davidii* var. *unicolor*.

Gene Name	Gene ID	Number of Amino Acids	Instability Index	Isoelectric Point	Aliphatic Index	Grand Average of Hydropathicity	Subcellular Localization
*LdARF1*	Lily01G01220.1	1124	73.4	6.29	66.65	−0.75	nucl
*LdARF2*	Lily01G30330.1	532	58.67	9.61	81.86	−0.286	nucl; vacu; golg
*LdARF3*	Lily01G30920.1	575	46.66	8.28	87.17	−0.273	chlo
*LdARF4*	Lily01G47960.1	758	51.26	5.95	73.39	−0.431	nucl
*LdARF5*	Lily02G49310.1	912	62.07	5.94	70.89	−0.515	nucl
*LdARF6*	Lily03G34540.1	635	48.39	7.61	75.78	−0.478	nucl
*LdARF7*	Lily03G39660.1	569	54.9	8.53	73.67	−0.294	nucl
*LdARF8*	Lily04G22850.1	905	71.27	5.78	76.44	−0.489	nucl
*LdARF9*	Lily04G65590.1	1011	60.35	5.16	73.66	−0.448	nucl
*LdARF10*	Lily04G65800.1	1010	58.3	5.25	73.17	−0.442	nucl
*LdARF11*	Lily05G26760.1	709	58.36	6.31	66.28	−0.53	nucl
*LdARF12*	Lily05G29210.1	418	44.8	6.03	78.11	−0.165	chlo
*LdARF13*	Lily05G29250.1	400	47.5	5.95	76.25	−0.169	cyto
*LdARF14*	Lily05G53910.1	687	47.73	6.82	80.86	−0.258	ER
*LdARF15*	Lily06G62050.1	370	53.95	9.81	76.7	−0.232	chlo
*LdARF16*	Lily07G19690.1	827	59.88	6.1	65.77	−0.621	nucl
*LdARF17*	Lily07G32300.1	874	65.87	5.84	74.83	−0.455	nucl
*LdARF18*	Lily08G16240.1	667	55.66	6.08	69.7	−0.582	nucl
*LdARF19*	Lily08G65450.1	1144	63.35	6.08	73.77	−0.616	nucl
*LdARF20*	Lily09G10990.1	853	72.34	6.04	74.2	−0.535	nucl
*LdARF21*	Lily10G00240.1	710	49.84	6.43	68.94	−0.37	nucl
*LdARF22*	Lily11G09810.1	739	55.73	6.62	71.8	−0.429	nucl
*LdARF23*	Lily11G12430.1	688	47.87	6.49	74.04	−0.461	pero
*LdARF24*	Lily12G41800.1	538	52.32	8.71	75.61	−0.271	ER

## Data Availability

Data supporting the findings of this study are available within the article and its Supplementary Materials. All primers used in this study are provided in Supplementary Table S1. The RNA-seq datasets generated and analyzed in this study have been deposited in the National Genomics Data Center (NGDC) under accession numbers PRJCA060256 and PRJCA068453, corresponding to the scale-derived bulblet regeneration transcriptome dataset and the tissue transcriptome dataset from different organs of *Lilium* ‘Siberia’, respectively.

## Supplementary Materials

The following supporting information can be downloaded at https://www.mdpi.com/article/10.3390/plants15172581/s1, Figure S1: The distribution of *LdARF* genes on chromosomes; Figure S2: Chromosomal localization, gene density distribution, and synteny relationships of the *LdARF* gene family in *Lilium davidii* var. *unicolor*. Figure S3: Analysis of cis-acting regulatory elements in the promoter regions of *LdARF* genes in *Lilium davidii* var. *unicolor*; Figure S4: Expression patterns of *LdARF17* during bulblet regeneration in *Lilium davidii* var. *unicolor*. Figure S5: Validation of TRV infection and relative expression of *LdARF3*, *LdARF9*, and *LdARF21* in VIGS-treated *Lilium davidii* var. *unicolor* scales; Figure S6: Molecular confirmation of transient transformation in *Lilium davidii* var. *unicolor* scales; Figure S7: Melting curve analysis of qRT-PCR products for *LdARF* genes; Table S1: The primers used in this study; Table S2: Summary of ‘Siberia’ tissue transcriptome reads mapped to the *Lilium davidii* var. *unicolor* reference genome. Table S3: Summary of ‘Siberia’ scale-derived bulblet regeneration transcriptome reads mapped to the *Lilium davidii* var. *unicolor* reference genome. Table S4: Pearson correlation analysis of *LdARF* expression profiles between ‘Siberia’ lily RNA-seq data and *Lilium davidii* var. *unicolor* qRT-PCR data during bulblet regeneration. Table S5: The function of *LdARF17* in the scale-induced bulblet regeneration verified by virus-mediated gene silencing technology; Table S6: Overexpression of *LdARF17* promotes scale-induced bulblet regeneration; Table S7: The relative expression of *LdARF* family members during different bulblet development stages (S0, S1, S2, S3 and S4) derived from the scaling propagation method; Table S8: The relative expression of *LdARF17*, bulblet regeneration-related genes and auxin-related genes in TRV2 and TRV2-*LdARF17* lines; Table S9: The relative expression of *LdARF17*, bulblet regeneration-related genes and auxin-related genes in EV and *LdARF17*-OE lines.

## Abbreviations

The following abbreviations are used in this manuscript:

ARF	Auxin Response Factor
Aux/IAA	Auxin/Indole-3-Acetic Acid
CDS	Coding Sequence
LTR	Long Terminal Repeat
NJ	Neighbor-Joining
qRT-PCR	Quantitative Real-Time Polymerase Chain Reaction
RNA-seq	RNA Sequencing
RNAi	RNA Interference
TF	Transcription Factor
TRV	Tobacco Rattle Virus
VIGS	Virus-Induced Gene Silencing
GUS	β-Glucuronidase
Hyg	Hygromycin resistance gene
HMM	Hidden Markov Model
BLASTP	Basic Local Alignment Search Tool for Protein sequences
MEGA	Molecular Evolutionary Genetics Analysis
CDD	Conserved Domain Database
MEME	Multiple Em for Motif Elicitation
Ka/Ks	Non-synonymous substitution rate/Synonymous substitution rate
