# Supplementary material for: Genome-Wide Identification of the LdARF Gene Family in Lilium davidii var. unicolor and Transient Functional Analysis of LdARF17 in Bulblet Regeneration
Source: Plants (Basel). 2026 Aug 24;15(17):2581. doi: 10.3390/plants15172581 (PMC13567442; doi:10.3390/plants15172581)
Supplement: Supplementary file 1 [file plants-15-02581-s001.zip › Figure S1.pdf]

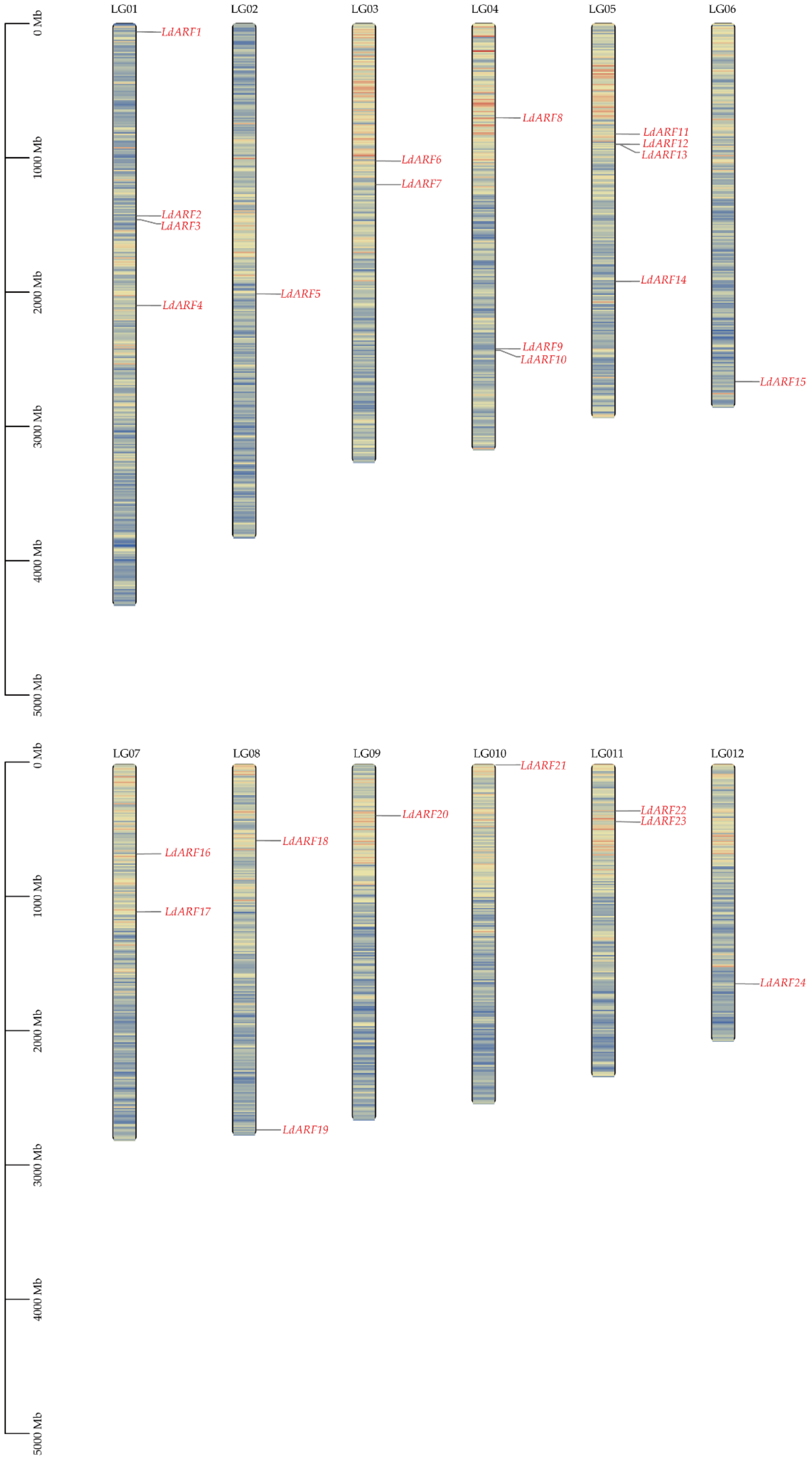

Figure S1. The distribution of *LdARF* genes on chromosomes.
