# Supplementary material for: Genome-Wide Identification of the LdARF Gene Family in Lilium davidii var. unicolor and Transient Functional Analysis of LdARF17 in Bulblet Regeneration
Source: Plants (Basel). 2026 Aug 24;15(17):2581. doi: 10.3390/plants15172581 (PMC13567442; doi:10.3390/plants15172581)
Supplement: Supplementary file 1 [file plants-15-02581-s001.zip › Figure S2.pdf]

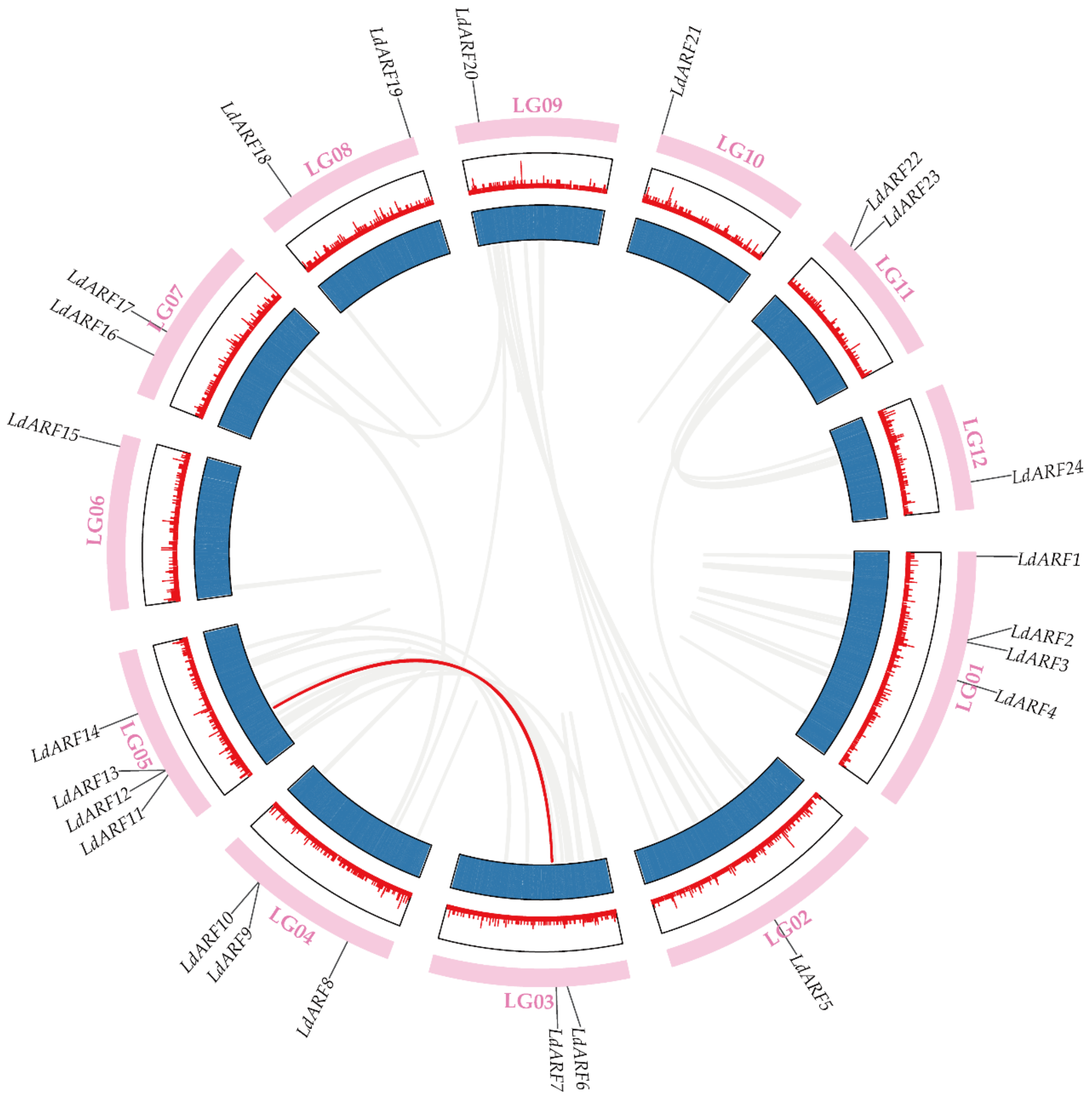

Figure S2. Chromosomal localization, gene density distribution, and synteny relationships of the *LdARF* gene family in *Lilium davidii* var. *unicolor*. The 24 *LdARF* members were mapped onto 12 linkage groups (LG01–LG12). The outermost circle indicates chromosome positions, with gene names shown at their corresponding loci. The blue bars represent chromosome segments, and the red histogram indicates gene density along chromosomes. Syntenic relationships among *LdARF* genes are displayed by gray lines, and the highlighted red line represents a duplicated gene pair identified within the genome.
