# Supplementary material for: Genome-Wide Identification of the LdARF Gene Family in Lilium davidii var. unicolor and Transient Functional Analysis of LdARF17 in Bulblet Regeneration
Source: Plants (Basel). 2026 Aug 24;15(17):2581. doi: 10.3390/plants15172581 (PMC13567442; doi:10.3390/plants15172581)
Supplement: Supplementary file 1 [file plants-15-02581-s001.zip › Figure S3.pdf]

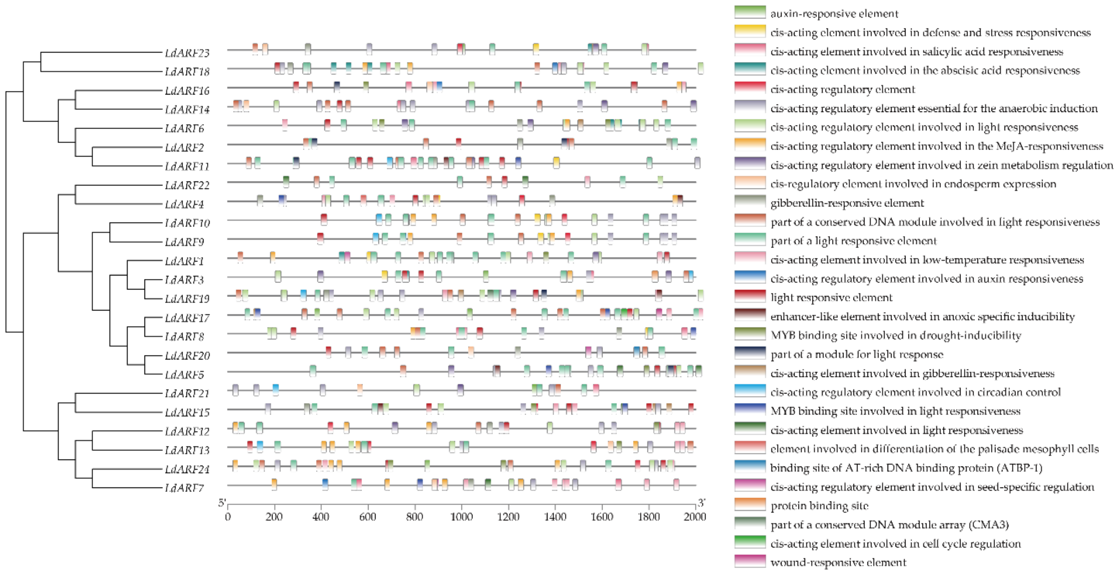

Figure S3. Analysis of cis-acting regulatory elements in the promoter regions of *LdARF* genes in *Lilium davidii* var. *unicolor*.
