# Supplementary material for: Genome-Wide Identification of the LdARF Gene Family in Lilium davidii var. unicolor and Transient Functional Analysis of LdARF17 in Bulblet Regeneration
Source: Plants (Basel). 2026 Aug 24;15(17):2581. doi: 10.3390/plants15172581 (PMC13567442; doi:10.3390/plants15172581)
Supplement: Supplementary file 1 [file plants-15-02581-s001.zip › Figure S4.pdf]

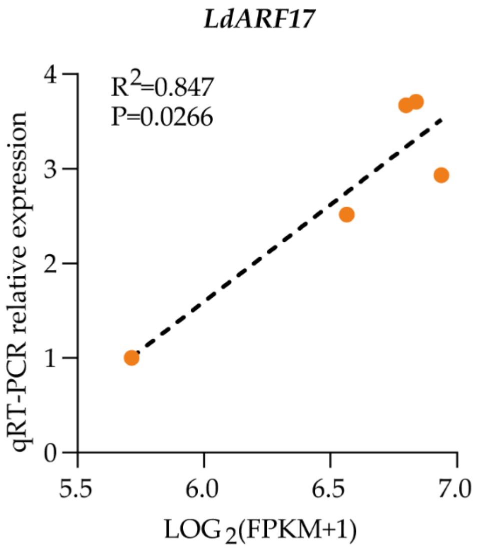

Figure S4. Expression pattern of *LdARF17* during bulblet regeneration in *Lilium davidii* var. *unicolor*. The expression profile of *LdARF17* was analyzed using RNA-seq data and validated by qRT-PCR across five regeneration stages (S0–S4). RNA-seq expression values were derived from 'Siberia' lily and are presented as $\log_2$(FPKM+1)-transformed values. qRT-PCR analysis was performed using *L. davidii* var. *unicolor* materials, and relative expression levels were calculated using the $2^{-\Delta\Delta Ct}$ method.
