# Supplementary material for: Genome-Wide Identification of the LdARF Gene Family in Lilium davidii var. unicolor and Transient Functional Analysis of LdARF17 in Bulblet Regeneration
Source: Plants (Basel). 2026 Aug 24;15(17):2581. doi: 10.3390/plants15172581 (PMC13567442; doi:10.3390/plants15172581)
Supplement: Supplementary file 1 [file plants-15-02581-s001.zip › Figure S5.pdf]

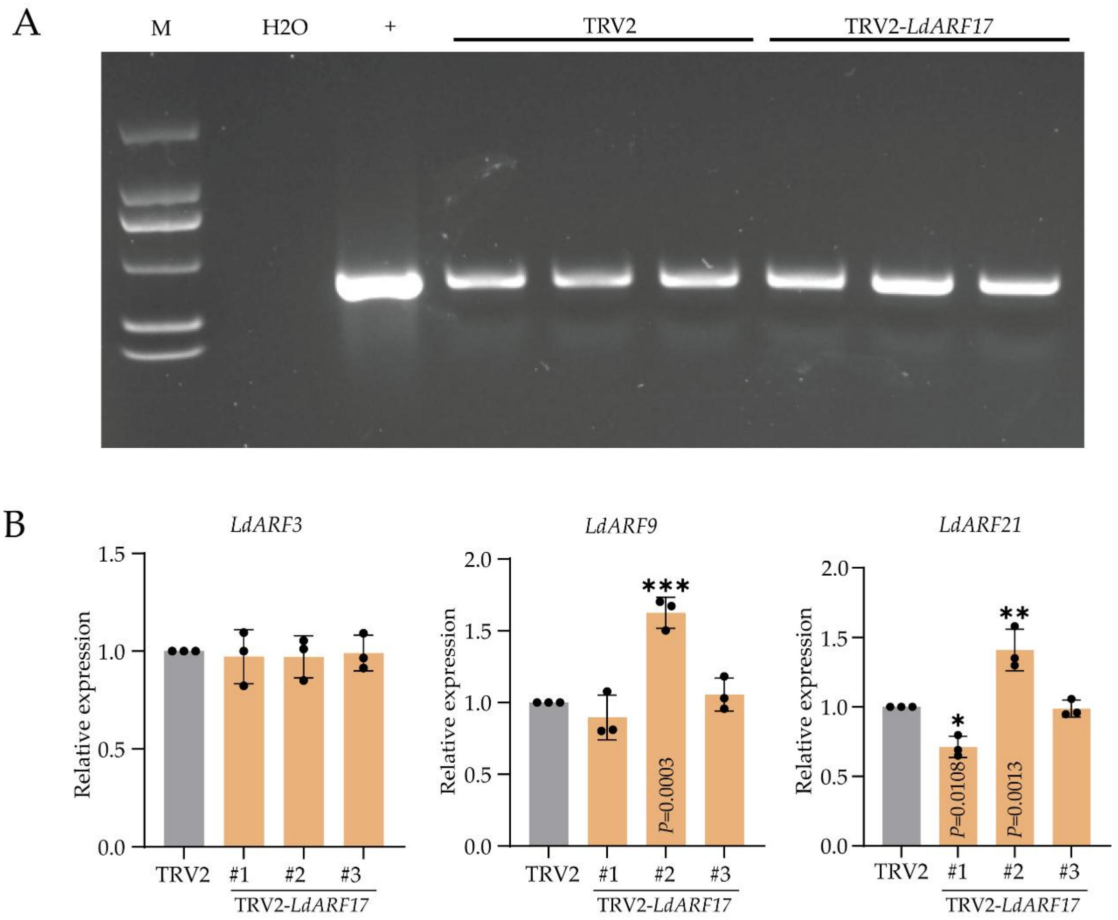

Figure S5. Validation of TRV infection and relative expression of *LdARF3*, *LdARF9*, and *LdARF21* in VIGS-treated *Lilium davidii* var. *unicolor* scales. (A) PCR detection of the TRV coat-protein (CP) gene in lily scale tissues. M, DNA marker; H_2O, negative control; +, positive control; TRV2, empty-vector control; TRV2-*LdARF17*, TRV silencing construct targeting *LdARF17*. Amplification of the expected CP band verified successful TRV viral infection in inoculated lily scales. (B) Relative expression levels of *LdARF3*, *LdARF9*, and *LdARF21* in TRV2 control and three independent TRV2-*LdARF17* silencing lines (n=3). Error bars represent standard error (SE). Statistical significance was determined using ordinary one-way ANOVA followed by Dunnett's multiple comparisons test compared with the TRV2 control. Asterisks indicate significant differences (*P<0.05, **P<0.01, ***P<0.001, ****P<0.0001).
