# Supplementary material for: Genome-Wide Identification of the LdARF Gene Family in Lilium davidii var. unicolor and Transient Functional Analysis of LdARF17 in Bulblet Regeneration
Source: Plants (Basel). 2026 Aug 24;15(17):2581. doi: 10.3390/plants15172581 (PMC13567442; doi:10.3390/plants15172581)
Supplement: Supplementary file 1 [file plants-15-02581-s001.zip › Figure S6.pdf]

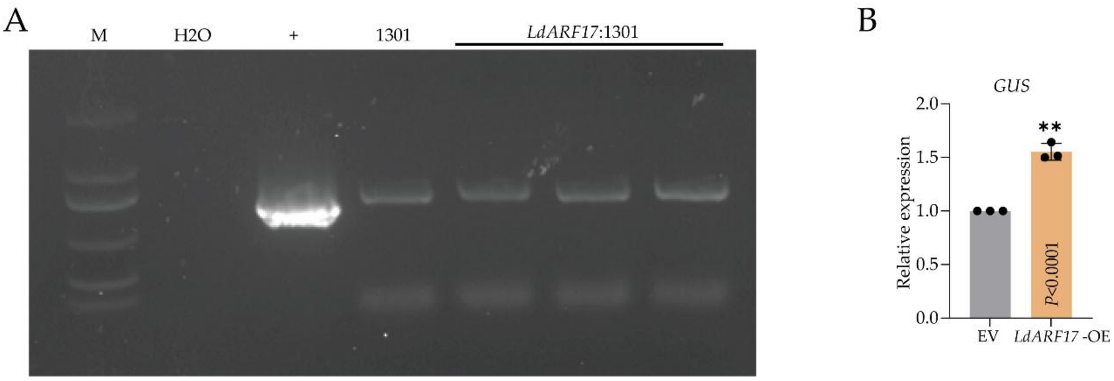

Figure S6. Molecular confirmation of transient transformation in *Lilium davidii* var. *unicolor* scales. (A) PCR-based detection of the hygromycin resistance (Hyg) gene in transiently transformed Lilium davidii var. unicolor scales overexpressing *LdARF17*. M, DNA marker; H_2O, negative control; +, positive control; 1301, empty vector control; *LdARF17*-OE, recombinant vector carrying *LdARF17*. (B) Relative expression analysis of the *GUS* gene by quantitative PCR (qPCR).The results confirm successful transformation and differential gene expression in lily scale tissues.
