# Supplementary material for: Genome-Wide Identification of the LdARF Gene Family in Lilium davidii var. unicolor and Transient Functional Analysis of LdARF17 in Bulblet Regeneration
Source: Plants (Basel). 2026 Aug 24;15(17):2581. doi: 10.3390/plants15172581 (PMC13567442; doi:10.3390/plants15172581)
Supplement: Supplementary file 1 [file plants-15-02581-s001.zip › Figure S7.pdf]

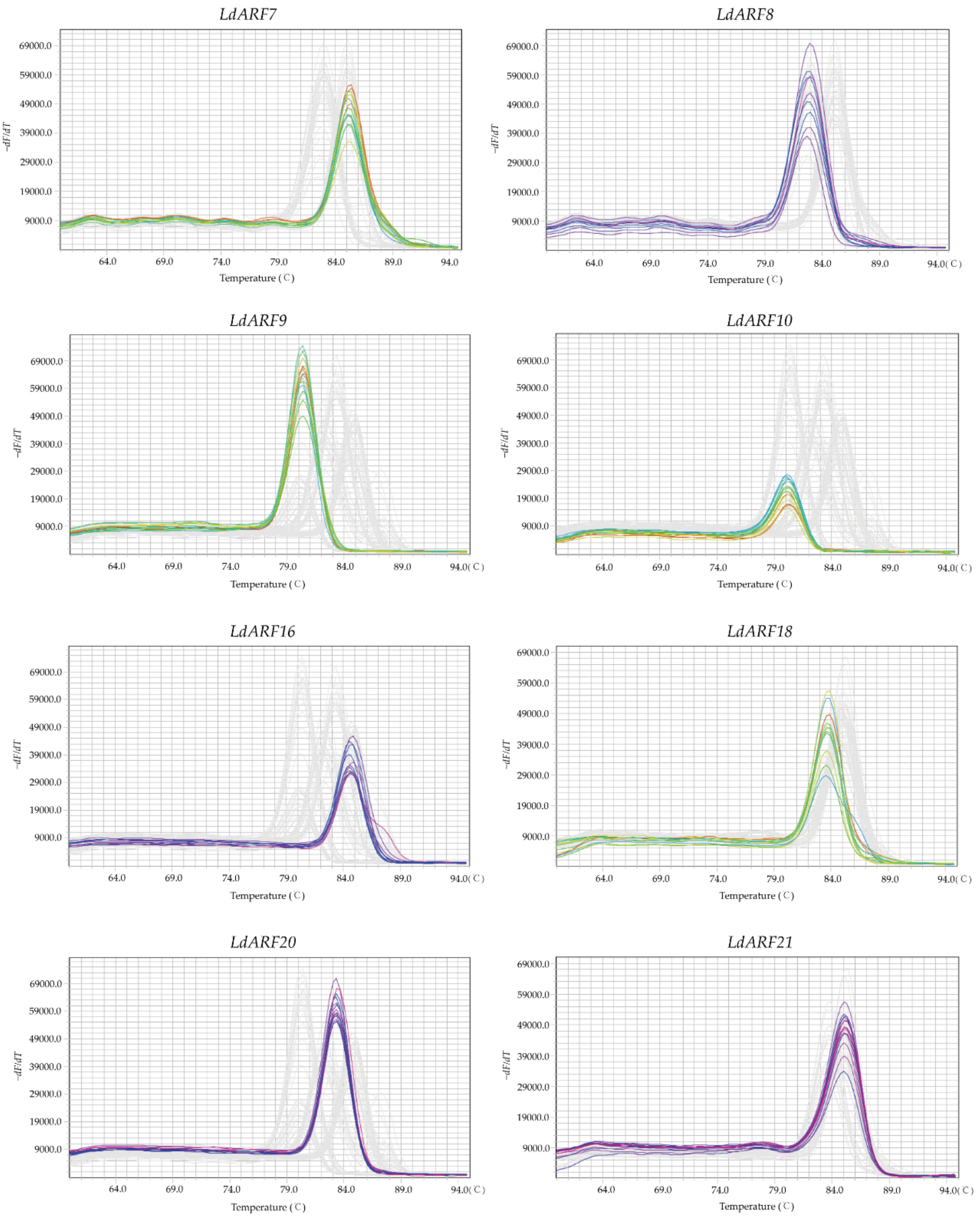

Figure S7. Melting curve analysis of qRT-PCR products for *LdARF* genes. Melting curve analysis was performed to evaluate the specificity of qRT-PCR amplification. The primer pairs used for *LdARF7*, *LdARF8*, *LdARF9*, *LdARF10*, *LdARF16*, *LdARF18*, *LdARF20*, and *LdARF21* generated single melting peaks, indicating specific amplification of the target transcripts without detectable non-specific products.
