# Supplementary material for: Genome-Wide Identification of the LdARF Gene Family in Lilium davidii var. unicolor and Transient Functional Analysis of LdARF17 in Bulblet Regeneration
Source: Plants (Basel). 2026 Aug 24;15(17):2581. doi: 10.3390/plants15172581 (PMC13567442; doi:10.3390/plants15172581)
Supplement: Supplementary file 1 [file plants-15-02581-s001.zip › Table S1.pdf]

Table S1. The primers used in this study.

Primer name	Sequence（5′-3′）	Applications
LdARF1-RT-F	ACGTTTCGGCACGTCTTTCG	RT-qPCR
LdARF1-RT-R	GAGAACTGAGTCGCCAGCCT	
LdARF3-RT-F	AAACAGTGGCGGAAGCAACG	
LdARF3-RT-R	GATTCTCCTCACCGGCGACA	
LdARF4-RT-F	CGGTAAAGGCGGCTGTAGAC	
LdARF4-RT-R	CGAGCTTCGAGAGGTCAACG	
LdARF5-RT-F	GGCAAGTCCTTCGGAGTTCG	
LdARF5-RT-R	CGGCGAACACTTGACTCCTC	
LdARF7-RT-F	GTACTGCGCCGACTCGATCT	
LdARF7-RT-R	TGGCGGAATTTCCACGGAGT	
LdARF8-RT-F	TGCTCGTAACCCGACTCAGAC	
LdARF8-RT-R	GCTGCTGCTGCTGCTGTATT	
LdARF9-RT-F	CGTAGAACTGCGGAAAAGTTA	
LdARF9-RT-R	CGAAATGTCCACAAGTTATCATA	
LdARF10-RT-F	ATCAGGTTTGAAAATTTTAA	
LdARF10-RT-R	CCAAGAACCGGTCATCATTG	
LdARF11-RT-F	TCCTCTGGTGACAGTGCCAA	
LdARF11-RT-R	GCCCTTGCTGAGTTGATGCT	
LdARF16-RT-F	TGAGCCAGCACAGGCGAATA	
LdARF16-RT-R	AGGTTCAGGCTGTGGATCGG	
LdARF17-RT-F	TCCAGAGCGGTGGAGTCAAT	
LdARF17-RT-R	TTGTGGATGCGTGCTTGACA	
LdARF18-RT-F	AGTGCCACACGTGTCTCCAA	
LdARF18-RT-R	GATCCACAGCACGACCGACT	
LdARF19-RT-F	AAGACACTGACTGCGAGCGA	
LdARF19-RT-R	CAGGCGGCTGCATTGAGAAG	
LdARF20-RT-F	GCCATCCAAGCGGTTAGCAG	
LdARF20-RT-R	GCAACAGACTGCTGTGCCAA	
LdARF21-RT-F	GGATGGGCGCAGTTAAGCAC	
LdARF21-RT-R	TTGTCGCTACTCGCATCCGT	
LdPIN1-RT-F	CGTGGCTCGCTTGACTTCAC	
LdPIN1-RT-R	GAGGACGACGATCTGCACCA	
LdLBD16-RT-F	TGCCCAATTTGCAGCCATCC	
LdLBD16-RT-R	CCTGGCTTGGGCTTGGTAGA	
LdLBD29-RT-F	TGCGGCCATCCATAAGGTGT	
LdLBD29-RT-R	GTCTTGAAGCCTCGCTTGGG	
LdWOX11-RT-F	TCCGCCGAAAGACGAGACAA	
LdWOX11-RT-R	AGCGAGATCGGCTTCAGCTT	
LdIAA14-RT-F	TTGGCCACCGGTTCGATCAT	
LdIAA14-RT-R	GGTGCGCCATCCATGCTAAC	

Primer name	Sequence（5′-3′）	Applications
LdACTIN-RT-F	ATCTATGAGGGTTATGCTCTTCC	
LdACTIN-RT-R	CATCAGGCAGCTCGTAACTTC	
GUS-RT-F	CTCTATGAACTCGAAGTCACAGC	
GUS-RT-R	GTAATGCGAGGTACGGTAGGAG	
LdARF17-F	ATGCGGCCCTTTTGCTTAA	Clone
LdARF17-R	TCAGTAATCCAGAGATCCCACAGA	
pcambia2300-LdARF17-F	ATGCGGCCCTTTTGCTTAA	Vector construction
pcambia2300-LdARF17-R	GTAATCCAGAGATCCCACAGACATG	
pCAMBIA 1301- LdARF17-F	ATGCGGCCCTTTTGCTTAA	
pCAMBIA 1301- LdARF17-R	GTAATCCAGAGATCCCACAGACATG	
ptrv2-LdARF17-F	TTGTATGGTGGAAGCGATGATATGG	
ptrv2-LdARF17-R	GACTGAGTGTGGTTATCCTGAATGG	
M13-F	ACTGGCCGTCGTTTTAC	Transgenic identification
M13-R	CAGGAAACAGCTATGAC	
TRV2-F	GCTGTTTGAGGGAAAAGTAGAGAAC	
TRV2-R	ACAAAAGACTTACCGATCAATCAAG	
Hyg-F	CTATTTCTTTGCCCTCGGACGAG	
Hyg-R	GAATCGGTCAATACACTACATGGC	
2300-GFP-F	ACAACCACTACCTGAGCACC	
